# The effects of inspiratory muscle training on physical function in critically ill adults: Protocol for a systematic review and meta-analysis

**DOI:** 10.1371/journal.pone.0300605

**Published:** 2024-03-22

**Authors:** Christopher Farley, Dina Brooks, Anastasia N. L. Newman

**Affiliations:** 1 Faculty of Health Science, School of Rehabilitation Science, McMaster University, Hamilton, ON, Canada; 2 Department of Respiratory Medicine, West Park Healthcare Centre, Toronto, ON, Canada; 3 Faculty of Medicine, Department of Physical Therapy, University of Toronto, Toronto, ON, Canada; 4 Rehabilitation Sciences Institute, School of Graduate Studies, University of Toronto, Toronto, ON, Canada; 5 Faculty of Medicine, Department of Medicine, University of Toronto, Toronto, ON, Canada; University of Sharjah, UNITED ARAB EMIRATES

## Abstract

**Introduction:**

Inspiratory muscle training (IMT) is one possible strategy to ameliorate respiratory muscle weakness due to invasive mechanical ventilation. Recent systematic reviews have focused on respiratory outcomes with minimal attention to physical function. The newest systematic review searched the literature until September 2017 and a recent preliminary search identified 5 new randomized controlled trials focusing on IMT in critical care. As such, a new systematic review is warranted to summarize the current body of evidence and to investigate the effect of IMT on physical function in critical care.

**Materials and methods:**

We will search for three main concepts (“critical illness”, “inspiratory muscle training”, “RCT”) across six databases from their inception (MEDLINE, EMBASE, Emcare, AMED, CINAHL, CENTRAL) and ClinicalTrials.gov. Two reviewers will independently screen titles, abstracts, and full texts for eligibility using the Covidence web-based software. Eligible studies must include: (1) adult (≥18 years) patients admitted to the intensive care unit (ICU) who required invasive mechanical ventilation for ≥24 hours, (2) an IMT intervention using a threshold device with the goal of improving inspiratory muscle strength, with or without usual care, and (3) randomized controlled trial design. The primary outcome of interest will be physical function. We will use the Cochrane Risk of Bias Tools (ROB2) and will assess the quality of the evidence using the Grading of Recommendations, Assessment, Development and Evaluations (GRADE) tool. This protocol has been reported according to the Preferred Reporting Items for Systematic Reviews and Meta-Analyses Protocols (PRISMA- P) guidelines and is registered with the International Prospective Register of Systematic Reviews (PROSPERO).

**Conclusion:**

Results will summarize the body of evidence of the effect of IMT on physical function in critically ill patients. We will submit our findings to a peer-reviewed journal and share our results at conferences.

## Introduction

Admission to an intensive care unit (ICU) often requires the initiation of invasive mechanical ventilation (IMV), either via an endotracheal tube or a tracheostomy, as a means of managing respiratory failure [1,2]. The introduction of positive pressure ventilation in the 1950s, at the height of the polio epidemic in Europe, modernized the provision of critical care and has led to a significant improvement in survival rates of patients admitted to ICU in the last seven decades [2]. Unfortunately, while IMV is a lifesaving intervention, it is also associated with the development of impaired respiratory muscle strength and endurance [2]. Diaphragmatic weakness (DW), defined as a decrease in diaphragm strength after the initiation of IMV, is associated with reduced pressure generating capacity and decreased diaphragm thickness [3]. It is estimated that diaphragmatic atrophy can begin within the first 18 hours of IMV and can impact up to 80 percent of patients admitted to ICU [2,4,5]. Alterations in diaphragm muscle thickness is also purported to occur more rapidly with controlled ventilator modes in comparison to spontaneous, patient-driven modes [6]. Patients diagnosed with DW are at greater risk of protracted intubations, failed extubations, extended critical care stays, and poor clinical outcomes [7]. In a 2016 prospective cohort study, reduced respiratory muscle strength, as measured by maximal inspiratory pressures (MIP), was an independent risk factor for one-year mortality in mechanically ventilated patients as compared to patients with intact respiratory muscle function (p = 0.007) [8]. These results were replicated in another 2016 prospective, 6-month observational cohort study, wherein mortality was higher in patients with diaphragm dysfunction (p = 0.04), as was duration of IMV [4].

Given the prevalence of DW and its impact on patient outcomes, strategies to remediate respiratory muscle strength during ICU admissions are important. Inspiratory muscle training (IMT) is one potential intervention that can be implemented in the ICU to help mitigate the effects of prolonged intubation. It involves targeted strengthening of the diaphragm and accessory inspiratory muscles through the application of external resistance during inspiration with the goals of improving muscular strength and endurance and decreasing shortness of breath [7,9]. Critically ill patients may present with a variety of signs or symptoms of respiratory weakness, including decreased chest expansion, dyspnea, decreased breath sounds, paradoxical breathing pattern, decreased lung volumes, difficulty weaning, and failed extubations [9]. Previous studies have shown that IMT can improve respiratory muscle function in patients admitted to critical care [10]. The use of a threshold inspiratory device to initiate progressive IMT significantly improved weaning success in a population of critically ill patients with failure to wean as compared to the control group who received usual care [11]. The introduction of a two-week course of IMT 48 hours post extubation led to statistically significant improvements in inspiratory muscle strength (p = 0.02), as measured by MIP, as compared to usual care [10]. Previous systematic reviews have also supported the use of IMT in the ICU, reporting significant improvements in both inspiratory muscle strength and duration of weaning [7,12], weaning success [12], and ICU length of stay [12]. These studies have focused primarily on respiratory outcomes, however, recent literature suggests there is a moderately positive correlation between increased MIP and physical function outcomes [13].

Three previous systematic reviews have been conducted since 2011 on the use of IMT with critically ill populations [7,12,14]. The initial review in 2011 [14] included three randomized controlled trials (RCT) and the subsequent reviews in 2015 and 2018 have included 10 [12] and 28 [7] trials respectively. This expansion in qualifying studies highlights the increasing use of IMT within the ICU. A recent preliminary search identified 5 additional RCTs since the last systematic review was conducted [7]. Given this increase in studies, it is necessary to summarize the current literature of IMT in critical care.

The aim of this systematic review is to understand the physical function outcomes for patients in the ICU who undergo IMT. To this end, the proposed review will strive to answer the following question: In adults admitted to ICU who required IMV for ≥24 hours, does IMT with a threshold device compared to usual care improve physical function? Consistent with reporting guidelines and to optimize the transparency of our conduct, we have developed this protocol [15].

## Materials and methods

This protocol is reported according to the Preferred Reporting Items for Systematic Review and Meta-Analysis (PRISMA) protocols (PRISMA-P) 2015 statement [15,16]. In accordance with the guidelines, our systematic review protocol was registered with the International Prospective Register of Systematic Reviews (PROSPERO) on 25 August 2023, the last day it was updated (registration number CRD42023451809). If amendments to this protocol are warranted, we will provide the date, a description, and rationale for each change. Our review will be conducted using the Cochrane methodology [17] and reported according to the PRISMA 2020 statement [18,19].

### Eligibility criteria

Studies will be eligible for inclusion if they enrolled adult (≥18 years) patients admitted to ICU who required IMV for ≥24 hours (Table 1). We required ≥24 hours of mechanical ventilation because of its association with skeletal muscle atrophy and respiratory muscle weakness [20,21]. A study which enrolled patients with and without IMV would be eligible if it reported our outcomes of interest separately for the IMV group. The intervention of interest is IMT using a threshold device with the goal of improving inspiratory muscle strength, with or without usual care. We specified that the intervention could be with or without usual care to ensure all appropriate IMT studies were included, even if the authors did not specify the inclusion of usual care. Usual care, such as medical, nursing, and allied health care is standard practice, thus all interventions groups would be provided usual care, even if not specified by the study’s authors. To ensure potential studies aimed to improve inspiratory muscle strength, we will only include studies that assessed MIP [7]. Comparator group treatments may include usual care, as defined by the studies’ authors (e.g. sham-IMT, routine physiotherapy, t-piece weaning, etc.). The primary outcome of interest will be physical function, as measured by any validated performance-based outcome measure, such as the Physical Function Intensive Care Unit Scored [22], the Intensive Care Unit Mobility Scale [23], and the Functional Status Score for the Intensive Care Unit [24]. We will include RCTs published in peer-reviewed journals if they are reported in English, French or Portuguese.

**Table 1:** Eligibility criteria.

	Inclusion	Exclusion
Population	Adults (≥18 years) Admitted to ICU ≥24 hours of invasive mechanical ventilation	Admitted to long term respiratory care centers
Intervention	IMT with a device with goal of improving inspiratory muscle strength With or without usual care	IMT intervention with no assessment of maximal inspiratory strength; IMT as a component of a bundle care intervention; T-piece weaning
Comparator	Usual care	None
Outcomes	Primary outcome: • Physical function (e.g., Physical Function Intensive Care Unit Scored [22]; Intensive Care Unit Mobility Scale [23]; Functional Status Score for the Intensive Care Unit [24]) Secondary outcomes: • Mortality • Length of stay (hospital, ICU) • Time to liberation from mechanical ventilation • Reintubation rate • Dyspnea (e.g., Borg Rating of Perceived Exertion [25]) • Respiratory muscle strength (e.g., maximal inspiratory strength; maximal expiratory strength) • Respiratory endurance (e.g., Ventilatory Endurance Test [26]) Timepoints^a^: • ICU discharge • Hospital discharge • 3-, 6-, 12-month post discharge	N/A
Study design	Randomised controlled trial	Quasi-randomised controlled trial Cluster randomised controlled trial Crossover randomised controlled trial
Language	English, French, Portuguese	None

ICU = intensive care unit; IMT = inspiratory muscle training; N/A = not applicable.

^a^ Timepoints may be refined dependent on timepoints reported in included studies.

### Information sources

We will search MEDLINE (OVID interface, 1946 onwards), EMBASE (OVID interface, 1974 onwards), Emcare (OVID interface, 1995 onwards), AMED (OVID interface, 1985 onwards), CINAHL (EBSCOhost interface, 1981 onwards), and Cochrane Central Register of Controlled Trials (Cochrane library interface). We will also search for trial protocols through ClinicalTrials.gov. Reference lists of included studies will be reviewed to identify any potentially relevant reports not identified through our search. Additionally, the authors’ personal files will be searched for relevant studies.

### Search strategy

Search strategies were developed by the authors (CF and AN) in consultation with a health sciences librarian who had expertise in conducting systematic reviews. Search terms include medical subject headings (MeSH) and keywords for the three main concepts of interest: (1) critical illness, (2) inspiratory muscle training, and (3) RCT. The draft MEDLINE search strategy is included in Table 2. Once the MEDLINE search strategy is finalized, it will be adapted to the syntax and the MeSH terms of the other databases. The final search strategy will be published on PROSPERO. Search strategies will be limited to human subjects only.

**Table 2:** Draft MEDLINE search using OVID interface.

Concept	Search terms
Randomized controlled trial [27]	1. randomized controlled trial.pt. 2. controlled clinical trial.pt. 3. randomized.ab. 4. placebo.ab. 5. drug therapy.fs. 6. randomly.ab. 7. trial.ab. 8. groups.ab. 9. 1 or 2 or 3 or 4 or 5 or 6 or 7 or 8 10. exp animals/ not humans.sh. 11. 9 not 10
Critical illness	12. intensive care units/ or burn units/ or coronary care units/ or respiratory care units/ 13. (intensive care or burn unit* or coronary care unit* or respiratory care unit* or ICU or ICUs).mp. 14. Critical Illness/ 15. critical* ill*.mp. 16. Critical Care/ 17. critical care.mp. 18. airway management/ or airway extubation/ or intubation, intratracheal/ or respiration, artificial/ or ventilator weaning/ or tracheostomy/ 19. (airway management or extubat* or intubat* or ventilator* or mechanical* ventilat* or tracheostomy or artificial respiration).mp. 20. 12 or 13 or 14 or 15 or 16 or 17 or 18 or 19
Inspiratory muscle training	21. exercise therapy/ or endurance training/ or resistance training/ 22. exercise therapy or endurance training or resistance training).mp. 23. (muscle* training adj2 (inspiratory or respiratory)).mp. 24. (muscle* strength* adj2 (inspiratory or respiratory)).mp. 25. physical therapy modalities/ or exercise movement techniques/ or breathing exercises/ 26. (physical therap* or physiotherap* or exercise movement or breathing exercise*).mp. 27. 21 or 22 or 23 or 24 or 25 or 26
Combined concepts	28. 11 and 20 and 27
Limits	Humans only

### Study screening and extraction

Search results will be uploaded to Covidence systematic review software (2020, Veritas Health Innovation, Melbourne, VIC, Australia), an internet-based program, to facilitate collaboration between reviewers during the study selection and data extraction process. Study selection will be conducted in 2 screening stages: (1) title/abstract and (2) full text. Screening forms will be developed for each stage, with piloting of the forms conducted prior to initiating formal study selection. Calibration exercises will be conducted prior to each stage of screening using five to ten studies to ensure consistency between reviewers.

The review authors (CF and AN) will conduct both stages of study selection against the eligibility criteria, independently and in duplicate. We will resolve disagreements through discussion or, if required, with arbitration by a third reviewer (DB). Reasons for study exclusion at the full text stage will be recorded and presented in the PRSIMA diagram.

The review authors (CF and AN) will complete all data extraction independently and in duplicate, with discussion or arbitration by a third reviewer (DB) to resolve discrepancies. A standardized extraction template will be developed on the Covidence platform. We will extract from the parent publication, associated supplemental files and cited protocols; where conflicting information exists between reports, the information described in the parent publication will take precedence for extraction. The following information will be extracted from qualifying texts:

- Study identification (i.e., title, first author, corresponding author, corresponding author email address, journal title, country of origin, study design, funding sources)
- Participant characteristics (i.e., eligibility criteria, number enrolled, number with baseline characteristics reported, age, sex, severity of illness, comorbid conditions and/or comorbidity score [e.g., Charlson Comorbidity Index], length of stay, duration of IMV)
- Intervention characteristics (i.e., intervention description, session frequency, intensity, session duration, and type of intervention, treatment fidelity)
- Comparator characteristics (e.g., intervention description, session frequency, intensity, session duration, type of intervention, and treatment fidelity)
- Setting (i.e., ICU type, hospital type)
- Outcome(s) assessed (i.e., outcome measure, assessment timepoint)
- Mean (standard deviation), median (interquartile ranges), and number (percentage) for the outcomes of interest.

Where a study reports multiple measures which represent one of our outcomes of interest, we will prioritize extraction in two ways: by extracting the study’s primary outcome first and then extracting the most comprehensively reported item second (i.e., the measure with the largest proportion of enrolled participant data).

### Risk of bias assessment

Risk of bias will be assessed for each extracted outcome using the Cochrane tool for assessing risk of bias in randomised trials version 2 (RoB 2) [28]. The RoB 2 assesses the risk of bias associated with each of the following domains: the randomisation process, deviations from the intended interventions, missing outcome data, outcome measurement, and selective reporting. Each domain will be assessed as “low risk of bias”, “some concerns”, or “high risk of bias”. Risk of bias assessments will be completed independently and in duplicate, with discussion to resolve conflicts. A third reviewer will arbitrate as needed. Using tables and graphs, we will create visual representations to illustrate the risk of bias assessments across all included trials.

### Data analysis and synthesis

#### Measures of treatment effect

Dichotomous data will be reported as risk ratio (RR) with 95% confidence intervals (CI). Continuous data will be reported as the mean difference with 95% CIs when the same outcome measure was used to assess a particular outcome between studies. When similar outcome measures are used, data will be reported as the standardized mean difference with 95% CIs. All study related data will be made freely available on Open Science Framework at the time of study completion.

#### Dealing with missing data

Where incomplete data exists in included studies, we will attempt to contact the corresponding author for clarification via email up to a maximum of three times. If contact is unsuccessful, we will exclude the missing data from the analysis and provide a narrative summary of the available results.

#### Assessment of heterogeneity

Clinical heterogeneity will be assessed by considering population, intervention, comparator, and outcome characteristics. Statistical heterogeneity will be assessed by visual inspection of forest plots, with the Chi^2^ test (significance level: 0.1), and via the *I*^2^ statistic, where 0% to 40% would represent heterogeneity that might not be important, 30% to 60% may represent moderate heterogeneity, 50% to 90% may represent substantial heterogeneity, and 75% to 100% considerable heterogeneity) [29].

#### Assessment of reporting biases

We intend to assess publication bias using funnel plots if at least ten studies have reported on each outcome assessed. Outcome reporting bias will be assessed by comparing outcomes reported in the trial publication to the corresponding protocol if one was available.

#### Data synthesis

A descriptive summary of findings will be completed with information presented in text and tables. If data are clinically and statistically homogenous, we will conduct meta-analyses using a random-effects model. Review Manager (RevMan 5.4) will be used to combine and analyze each outcome. If data are significantly heterogeneous, we will complete a narrative summary of the results only.

#### Subgroup analysis

Analyses will be conducted to explore outcomes among the following subgroups:

- Time of IMT initiation (i.e., prior to versus after liberation from IMV)
- Duration of IMV (i.e., prolonged [≥ 96 hours] versus short-term [<96 hours]) [30]
- Treatment fidelity (i.e., high [≥80% planned dose] versus low [<80% planned dose]) [31]
- By diagnosis (i.e., COVID-19; neurological population such as stroke, head injury, spinal cord injury, Guillain-Barre syndrome)
- By age (i.e. ≥ 65 years versus <65 years)

#### Sensitivity analysis

There are no planned sensitivity analyses.

### Quality of evidence

The quality of evidence will be assessed using Grading of Recommendations, Assessment, Development and Evaluations (GRADE) [32]. Using GRADE, we will assess the quality of evidence across the following domains: risk of bias, inconsistency, indirectness, imprecision, and publication bias. GRADE assessments will be conducted independently and in duplicate. Disagreements will be resolved with discussion or, if needed, by a third reviewer.

### Dissemination of results

The resultant manuscript will be submitted for publication in a peer-reviewed journal and for presentation at a critical care-relevant conference.

## Conclusion

Diaphragmatic weakness can begin to develop within hours of initiating IMV [20]. The use of IMV for greater than 24 hours has been associated with both respiratory [20] and skeletal muscle [21] weakness which can have significant impacts on patient function, both during admission and post discharge. During the COVID-19 pandemic, Canada experienced a 22 percent increase in the use of IMV between January 2020 and September 2022 compared to the years 2013–2014 [33–36]. Interventions targeting the remediation of respiratory muscle function may improve functional outcomes for critically ill patients. Given the increased use of IMV during the recent pandemic, and the length of time between the last review, this systematic review will synthesize current literature and the results of this review may help guide evidence-based practice for critically ill patients at risk of DW.

## Supporting information

S1 File PRISMA-P checklist. (DOCX)
